# Targeting Programmed *Fusobacterium nucleatum* Fap2 for Colorectal Cancer Therapy

**DOI:** 10.3390/cancers11101592

**Published:** 2019-10-18

**Authors:** Kumar Ganesan, Songhe Guo, Sundaz Fayyaz, Ge Zhang, Baojun Xu

**Affiliations:** 1 Food Science and Technology Program, Beijing Normal University-Hong Kong Baptist University United International College, Zhuhai 519087, China; kumarg@hku.hk; 2 Department of Microbial and Biochemical Pharmacy, School of Pharmaceutical Sciences, Sun Yat-Sen University, Guangzhou 510006, China; guosonghe@126.com; 3 Laboratory for Translational Oncology and Personalized Medicine, Rashid Latif Medical College, Lahore 44000, Pakistan; sundas.khan23@yahoo.com

**Keywords:** *Fusobacterium nucleatum*, CRC, genetically programmed, cancer therapy

## Abstract

Colorectal patients generally have the maximum counts of *Fusobacterium nucleatum (F. nucleatum)* in tumors and elevate colorectal adenomas and carcinomas, which show the lowest rate of human survival. Hence, *F. nucleatum* is a diagnostic marker of colorectal cancer (CRC). Studies demonstrated that targeting fusobacterial Fap2 or polysaccharide of the host epithelium may decrease fusobacteria count in the CRC. Attenuated *F. nucleatum*-Fap2 prevents transmembrane signals and inhibits tumorigenesis inducing mechanisms. Hence, in this review, we hypothesized that application of genetically programmed fusobacterium can be skillful and thus reduce fusobacterium in the CRC. Genetically programmed *F. nucleatum* is a promising antitumor strategy.

## 1. Introduction

Colorectal cancer (CRC) is the third-highest widespread malignant neoplasm and the fourth extremely common cause of malignancy death globally, and the five-year existence rate is less than 65 percent [1]. It is accountable for 694,000 demises yearly worldwide. It is a multifaceted disease, caused by genetic predisposition, lifestyle (sedentary, smoking), or diet (alcohol or red and processed meat consumption), and environmental exposure to various xenobiotics that could result in CRC development. Gut microbiota seems to be the mediator of this causal relationship, being disturbed by the exposure to such compounds, thus causing tumorigenic phenomena, a fact that constitutes a novel topic of research [2]. The mortality rate of CRC is based on the availability of medical resources [3]. In recent years, accumulating evidence greatly suggested a relationship between gut microbiota composition and CRC [4,5,6]. The human intestinal tract contains more than 100 trillion microorganisms that play a great significant function in human health. These organisms regulate gut homeostasis by maintaining several biological activities, including mucosal barrier, metabolic and immune functions [3,7,8]. Any disturbances occur in gut equilibrium, it may cause various intestinal illnesses, including Crohn’s disease, ulcerative colitis, and colorectal neoplasms [9,10]. There is great evidence that disturbance of gut microbiota can also lead to various systemic diseases such as diabetes, obesity, cancer, cardiovascular, and central nervous system disorders [11,12]. Increasing evidence confirmed that the gut microbiota is extremely connected with colorectal neoplasms [3,13,14,15].

Numerous investigations have confirmed that the levels of *Bacteroides*, *Prevotella*, *Leptotrichia*, *Clostridium difficile*, *Streptococcus gallolyticus*, *Bacteroides fragilis*, *Enterococcus faecalis*, *Campylobacter* spp., *Escherichia coli*, *Fusobacterium nucleatum*, and *Streptococcus bovis* are considerably higher in CRC compared to those in nearby normal tissue [16,17,18,19]. Studies in fecal samples containing Hungatella hathewayi, *F. nucleatum*, *Clostridium asparagiforme*, *Klebsiella oxytoca*, *E. coli*, *Bilophila wadsworthia*, and the genera *Lactococcus*, *Porphyromonas*, *Odoribacter*, *Bilophila*, and *Pyramidobacter* found to be enriched in patients with CRC [20,21]. *F. nucleatum* is a Gram-negative anaerobic bacterium found to be considerably higher and synergistically associated with other Gram-negative bacteria that promote the incidence and pathogenesis of CRC [22,23,24]. Gathering evidence suggested that the tumor tissues and fecal specimens of CRC patients have significantly increased counts of *F. nucleatum* [25,26,27]. This higher count of the organism may contribute to the development of CRC [28,29]. In a recent review regarding the role of oral bacteria and intestinal dysbiosis in CRC, it has been indicated that F. nucleatum merely resembles a passenger rather than a driver of intestinal dysbiosis in CRC according to the “driver-passenger model” about microbiota-driven CRC pathogenesis [30]. Nevertheless, the exact role of this specific bacterium in CRC progression is still an ongoing debate. It would be important at this point to include a brief summary of the “driver-passenger” model of microbiota dysbiosis, as introduced by Tjalsma et al. [31] since it is a crucial pathogenic concept regarding CRC. Hence, the F. nucleatum is a diagnostic marker of CRC. The positive detection rates of *F. nucleatum* in CRC patients testified by diverse study groups listed in Table 1. Further investigations have also confirmed that a higher count of *F. nucleatum* in CRC related to shorter survival rates [26,29].

**Figure 1:**
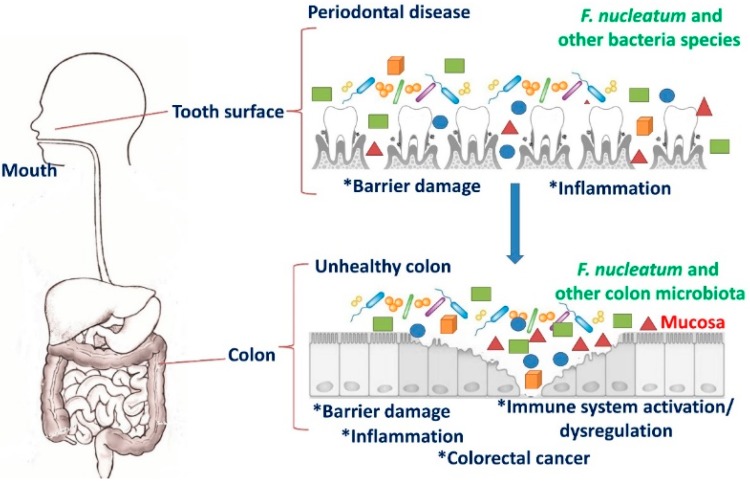
Migration of *F. nucleatum* from the oral cavity to colon that promote colorectal cancer (CRC).

*F. nucleatum* normally lives in the oral cavity of human and is commonly associated with diseases such as gingivitis, periodontal plaque, tonsillitis, sinusitis, chronic periodontitis, liver abscess, and appendicitis [19,24,50]. In addition to oral infections, this organism has been implicated in respiratory tract infections, cardiovascular disease, arthritis, Alzheimer’s disease, adverse pregnancy outcomes, and the development of various cancers including esophageal, gastric, and colon [51]. It exhibits high levels of homology with other *Fusobacterium* species including, *F. alocis*, *F. periodonticum*, and *F. simiae*. All these *Fusobacterium* species reside in oral cavities [51]. It is usually absent or habitually not found elsewhere in the body under normal conditions [52]. Until recently, *F. nucleatum* was thought to primarily be a component of the human oral microbiota and only an occasional resident of the gut. However, this premise was built on a culture-based examination of stool, which usually does not contain high numbers of live, epithelium-associated bacteria. FISH was used to elegantly demonstrate an association between invasive *Fusobacterium* spp. (including *F. nucleatum*) cells with inflamed appendix tissues, suggesting that the gut could be a hitherto unrecognized niche for this pathogen [53]. The mechanism of migration of these bacteria from the oral cavity to intestines to promote CRC are illustrated in Figure 1.

CRC constitutes a huge global economic problem and vigorous action should be taken to reduce the financial cost of this disease [54]. CRC is one of the leading and genetically categorized malignancies, with definite somatic mutations, oncogenes, and tumor suppressor genes. These mutations and other cellular regulators are essential for the development of adenomatous lesions to hostile carcinoma [25,55]. Accumulating evidence showed that *F. nucleatum* is among the most abundant species of bacteria in CRC tissues [43,56]. The outer membrane of this organism contains variable thickness of lipopolysaccharide (LPS) that may present pathogenic properties [52], and are vital for the evasion of the immune system in the human [50].

In recent years, *F. nucleatum* has been recognized to be a possible causative agent of CRC, tumor development and promotes colorectal tumorigenesis in *Apc*^*min*/+^ mice [29,57,58]. *In situ* hybridization studies have also confirmed that *F. nucleatum* is largely connected with malignancy cells in the metastatic lesions [58]. It stimulates tumor cell proliferation in CRC by activating β-catenin signaling and stimulating upstream regulation of oncogenic gene expression *via* the adhesive membrane virulence factor, includes protein adhesins, toxins, and enzymes [28]. Previously, Fecal *F. nucleatum* infection has been recognized as a significant diagnostic marker for CRC [47]. Our recent study also confirmed that among the important probiotics-*Faecalibacterium* and *Bifidobacterium*, *F. nucleatum* has identified as biomarkers for early CRC screening [59]. Taken together, these investigations demonstrated that *F. nucleatum* plays a significant role in the prime causes, diagnostic markers and progression of CRC and development.

## 2. *F. nucleatum* Mediate CRC and Inhibits Host Immune Response

*F. nucleatum* has been linked to immune suppression, through the promotion of lymphocytic apoptosis [60], and the abundance of *F. nucleatum* has been found to be inversely proportional to cluster of differentiation 3 (CD3^+^) T-cell density [61]. Recently, growing evidence demonstrated a high relationship between the infection of *F. nucleatum* and various cancers thus proposing innovative approaches in malignancy prevention by targeting *F. nucleatum* [29,56]. The earlier investigation has also established that *F. nucleatum* induces a noteworthy humoral immune response in chronic oral infection [62,63]. Recently, in our lab, we confirmed that *F. nucleatum* infection provoked high-level serum antibodies to *F. nucleatum* in CRC patients [64]. Using the sera of CRC patients to probe the bacteria protein extract, we found a robust reactive antigen, alkyl hydroperoxide reductase subunit C, activates the anti- *F. nucleatum* immune response [64].

*F. nucleatum* is a facultative intracellular anaerobic microorganism, possibly activating, proliferating, and migrating macrophages/monocytes that can provoke CRC development [34,65,66]. *F. nucleatum* can invade into endothelial and epithelial cells, induces the synthesis of pro-inflammatory cytokines, inflammatory lesions ultimately leading to CRC [29,67,68]. A contemporary study has also provided insights into the connection between the gut microbiota and the capacity of inflammatory cytokine production [69]. *F. nucleatum* promotes local inflammation and elevates the expression of inflammatory cytokines (Interleukins-IL-6, IL-8, tumor necrosis factor-alpha (TNF-α), and cyclooxygenase (COX-2), contributing to tumorigenic effects in CRC [28,29,69]. It can also induce chemokine C-C-motif ligand 20 (CCL20) expression in CRC while they are treated with *F. nucleatum* [66].

Nuclear factor kappa B (NF-κB) is a transcription factor that participates in regulating many gene expressions and promoting tumor development and progression [70]. It is well recognized as having crucial relationship with inflammation and cancer. The enrichment of the *F. nucleatum* triggers NF-κB activation that can be significantly involved in CRC development [71]. The expression of NF-κB is quite often triggering in *F. nucleatum* -enriched CRC [27,29]. Consistent with this study, Rubinstein et al. [28] have also confirmed that wild-type *F. nucleatum* continuously induces the expression of NF-κB in a human colon tumor (HCT116) xenograft model. Recently, two outer membrane proteins from *F. nucleatum* have been taken for attention, namely, fibroblast activation protein 2 (Fap2) and Fusobacterium adhesin A (FadA). Fap2 is a galactose-sensitive hemagglutinin and adhesive protein, which contributes to the invasive ability of *F. nucleatum* into the human cells that bind to D-galactose-β (1-3)-N-acetyl-D-galactosamine (Gal-GalNAc) [72,73,74,75]. FadA is another surface adhesive protein, exclusively present in *F. nucleatum* that plays a vital function in the mechanism of cell–cell attachment [75]. These two described proteins have greatly participated in the host cell attachment that modulates the expression of CRC signaling [28,76].

Yang et al. [27] found the massive quantities of *F. nucleatum* in the CRC tissues and the team further observed the increased invasion rate, proliferation, and xenograft tumors in humanized mice. *F. nucleatum* (specifically LPS) binds to TLR4, which trough myeloid differentiation primary response gene 88 (MYD88) signaling activates the NF-κΒ pathway, which enhances the gene expression of miR-21 [30]. Toll like receptor 4 (TLR4) is a chief receptor for bacterial LPS that overexpresses in CRC and plays a vital function in tumor development [77]. miR-21 may serve as a key promotor of colitis-associated colon cancer [78,79]. Earlier studies revealed that patients with a higher count of *F. nucleatum* and miR-21 exhibited the lowest survival rate [27]. Antibiotic treatment of CRC in mice bearing xenograft models reduced the count of *Fusobacterium* and prevent the proliferation of all tumor growth. However, these findings debate with the clinical investigation of antimicrobial interventions as effective management with *F. nucleatum* -associated CRC [80]. Moreover, our study also identified that *F. nucleatum* is sensitive to the tryptophan-depleted microenvironments and kynurenine could inhibit the growth of *F. nucleatum*, suggesting that tryptophan metabolism plays a role during the infection [65]. In addition, our study further confirmed that the subunit vaccine for *F. nucleatum*, such as alkyl hydroperoxide reductase subunit C can reduce *F. nucleatum* load in the intestinal tract [59,81].

Gut microbiota of humans comprises a diverse range of microbial strains in which certain strains are well recognized as carcinogenic agents [76]. In 2012, two different studies confirmed that *Fusobacterium* species or *F. nucleatum* in specific is excessively habituated in CRC tissues when compared to the normal mucosa of the gut [25,58]. *Fusobacterium* species are normal inhabitants of the oral cavity and poor invader to the healthy intestine and however, during an unhealthy tumor environment, these organisms can be reached to the gut [75]. These outcomes were effectively confirmed by McCoy et al. [57] and these authors have demonstrated an excess of *Fusobacterium* species, acknowledged as CRC precursors, which are highly populated in the colorectal adenomas (CRAs) when compared to the normal gut mucosa. Subsequently, numerous investigations have also proved the connection between *Fusobacterium* and CRA [82]. To recognize the molecular pathway associated with *F. nucleatum*, inflammation, and CRC, Kostic et al. [29] have demonstrated using animals (ApcMin/+) and found the genetic vulnerability of emerging colonic cancers. These animals were administered with invasive organisms of *F. nucleatum* initially obtained from the patient’s intestine of inflammatory bowel disease. The outcome showed elevated tumorigenesis, caused due to elevating infiltrating cells of the myeloid lineage (dendritic cells, macrophages, and granulocytes) [29]. Eventually, *F. nucleatum* induced inflammation as well as tumor proliferation through regulating tumor-immune microenvironment [29]. These animal trials confirmed that human colonic strains strongly correlated with the richness of *F. nucleatum* and pro-inflammatory markers expression [29,57]. *F. nucleatum* has been known to suppress anti-tumor immunity by preventing tumor-killing cells (natural killer cells and tumor-infiltrating lymphocytes) [74]. Based on these experimental outcomes, *F. nucleatum* is not only inhabited and developed in CRAs and adenocarcinomas but also augments tumor progression and existence through the tumor-immune microenvironment.

## 3. Fap2 in *F. nucleatum* Mediates CRC through Host Gal-GalNAc

Fibroblast activation protein 2 (Fap2) plays a critical function in mediating CRC development through binding with acetylgalactosamine (Gal-GalNAc), which is overexpressed in human metastases and colorectal adenocarcinoma [83]. Fap2 is a galactose-sensitive hemagglutinin and adhesin that possibly plays a function in the virulence of *Fusobacterium*. It is a 390-KDa protein encoded by the *Fap2* gene of *F. nucleatum* [73]. The outer membrane composed of 3692 amino acid, which is recognized to stimulate apoptosis in human lymphocytes [48]. Earlier study demonstrated that Fap2 has participated in the binding of *F. nucleatum* to malignant cells and interacts with the immunoglobulin and immunoreceptor tyrosine based inhibitory motif (ITIM) domain receptor mainly expressed on natural killer cells (NK), regulatory T cells (Treg), cluster of differentiation-CD8^+^, and CD4^+^ T cells [75]. The binding of Fap2 to T cell immuneoreceptor with Immunoglobulin G (TIGIT) was found to inhibit the activity of NK cells against the tumor cells, thus causing the development of CRC [74].

Abed et al. [84] have confirmed the quantities of fusobacterial lectin and host polysaccharide that explicates fusobacterial abundance in the CRC. This study further explains that host epithelial Gal-GalNAc over-expressed in the CRC, which is identified by fusobacterial Fap2, providing its role as a Gal-GalNAc lectin [84]. High Gal-GalNAc levels are also found in CRC and are connected with fusobacterial genomic DNA occurrence in these metastases, representing the ability of fusobacteria to colonize CRC [85]. The team has also further confirmed in orthotopic rectal colon tumor (CT26) adenocarcinoma model that intravascular injection of oral *F. nucleatum* strain favors inhabiting in CRC tissue. This transmission route is mediated through the binding of Fap2 to Gal-GalNAc. Hence, all these supporting ideas have provided oral fusobacteria that may colonize CRC via a hematogenous route [84].

Gal-GalNAc lectin is universally familiar by the immune sera of patients with amoebic liver abscess and various other diseases [86]. It plays a crucial role in cytolysis and phagocytosis of human and rat colonic mucin glycoproteins. Earlier study related to the uptake of *L. pneumophila* by *H. vermiformis* was specifically inhibited by Gal-GalNAc against the lectin of *E. histolytica*. Remarkably, the inhibition of invasion by Gal-GalNAc was connected with inhibition of bacterial-induced tyrosine dephosphorylation of *H. vermiformis* proteins [87]. Normally, the functions of the lectin comprise the host cell binding, cytotoxicity, complement resistance, induction of encystation, and generation of the cyst wall. In addition, the functions of the lectin in both differentiation and virulence suggest that it may be a pivotal molecule that determines the severity of the infection from a commensal state resulting from increased encystation to an invasive state [88]. Earlier studies, high levels of the tumor expressed Gal-GalNAc moieties have shown in adenocarcinomas of various organs such as stomach [89], prostate [90], ovary [91], colon [92], uterus [93], pancreas [94], breast [89], lung [95], and esophagus [96].

Clinical fusobacteria strains that present lacking Fap2 or inactivated Fap2 mutants demonstrate reduced binding to Gal-GalNAc-expressing CRC cells [84]. Providing the tumorigenic role of fusobacteria and its immune evasion potential, the removal of fusobacteria might be a promising and improve treatment outcome of the CRC [85]. Moreover, fusobacteria seems to explicitly bind to Gal-GalNAc-displaying tumors, it must be programmed as a stage for the treating CRC. As immunosuppression is undesired in the future fusobacterial-based tumor therapy, the interesting novelty of detecting and inactivation of the Fap2 TIGIT receptor-activating domain highly required.

*F. nucleatum* infects the oral cavity and reaches the colon, whereby it causes tumor progression. However, the proposal of the programmed *F. nucleatum* promotes a healthy colon and prevents tumor prevention in the host. Outer membrane protein of *F. nucleatum*- Fap2 contributes to the invasive ability of *F. nucleatum* into the human that binds to E-cadherin and Gal-GalNAc. Fap2 binds only to Gal-GalNAc. FadA binds to E-cadherin [97]. Through Toll-like receptor 4, the *F. nucleatum* actively triggers the signals to MYD88 that stimulate activating protein-1 (AP1), NF-κB, mammalian target of rapamycin (mTOR), and extracellular signal-regulated kinase (ERK) pathways and eventually cause tumorigenesis. Although these facts are generally true, the activation of TLR4 is due to binding with LPS, not Fap2 [77]. The attenuated *F. nucleatum*-Fap2 prevents transmembrane signals and inhibit tumorigenesis inducing mechanisms. The genetically programmed mechanism can be achieved in fusobacterial Fap2 by the following steps: mutagenesis, rDNA techniques, attenuation of oncogenic materials performed by using vector encoded siRNA and shRNAs; induction of tumor suppressor genes, and immunogenic peptides. Targeting programmed fusobacterial Fap2 may reduce fusobacterium count in the CRC and promotes a healthy colon. Thus, the programmed *F. nucleatum* Fap2 is a promising anti-tumor activity against CRC.

**Figure 2:**
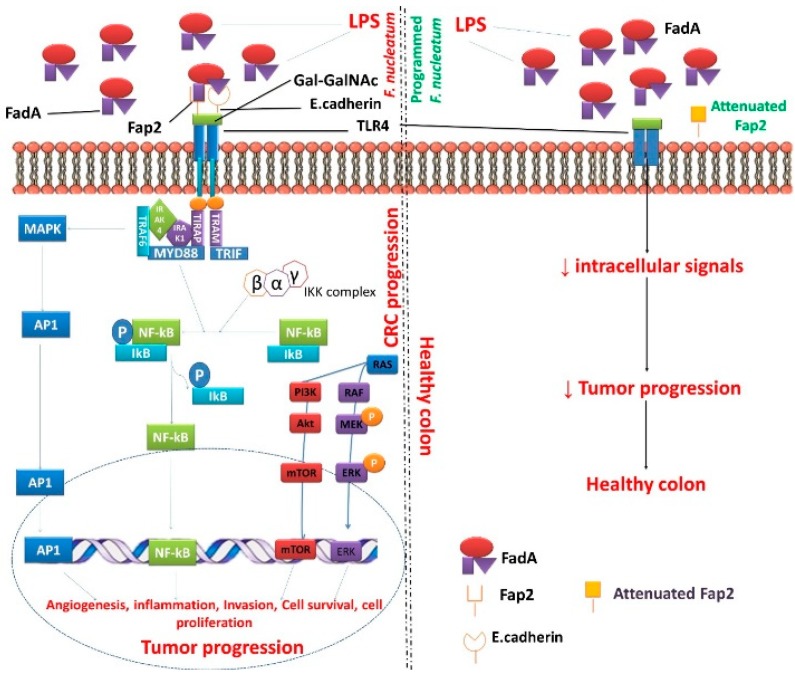
Programmed/attenuated *F. nucleatum* Fap2 as a potent factor for the treatment of CRC. Abbreviation: Akt-protein kinase B; Ap1-activating protein-1; ERK-extracellular signal-regulated kinase; FadA- Fusobacterium adhesin A; Fap2-fibroblast activation protein 2; Gal-GalNAc-acetylgalactosamine; IkB-Ikappa B proteins; IKK-Ikappa B kinase; IRAK1-Interleukin-1; receptor-associated kinase 1; IRAK4-Interleukin-1 receptor-associated kinase 4; LPS-lipopolysaccharide; MAPK-mitogen-activated protein kinase; MEK-MAPK-ERK-kinase; mTOR-mammalian target of rapamycin; MYD88-myeloid differentiation primary response gene 88; NF-kB-nuclear factor kappa-B; p-phosphorylated; PI3K-phosphatidylinositol 3-kinase; RAF-rapidly accelerated fibrosarcoma; RAS-rat sarcoma; TLR4-Toll-like receptor 4; TRAF6-Tumor necrosis factor receptor associated factor 6; TRAM-Transverse rectus abdominis myocutaneous; TRAP-thyroid hormone receptor associated protein.

Adhering to the gut epithelium by the various cell surface proteins, FadA, Fap2, and role of radiation genes (RadD) expressed by *F. nucleatum* can cause CRC in humans and produce inflammatory factors in the tumor microenvironment [98]. Rubinstein et al. [28] demonstrated that *F. nucleatum* enters into the host and persuades inflammation and oncogenic responses that proliferate CRC cells via its FadA attachment. FadA is a surface adhesive protein localized in *F. nucleatum* that functions as cell binding [75]. These FadA proteins fix with host E-cadherin (cellular adhesion) to trigger Wnt/*β-catenin* signaling pathways and regulate the inflammation as well as oncogenic responses (Figure 2). FadA interacts with E-cadherin at a locality of the 11-amino-acid region [28]. However, this amino acid proximately synthesizes a new peptide in the human, which terminates FadA induced cell proliferation, inflammation, and oncogenic responses in the colon [28]. In patients with adenomas and adenocarcinomas, FadA levels are 10–100 times higher than normal subjects [57]. These elevated FadA levels in CRC mostly associate with augmented inflammatory signaling and tumorigenic responses [28]. This investigation further reveals a key mechanism of *F. nucleatum* that can potentially regulate CRC and recognizes FadA as a possible diagnosis and therapeutic marker for CRC [76]. *F. nucleatum* binds to the patient’s adenocarcinomas associated with Gal-GalNAc expression that has been diminished upon O-glycanase treatment [84]. Therefore, targeting Fap2 in *F. nucleatum* and or host epithelial Gal-GalNAc could shorten fusobacteria potentiation in the CRC.

Researchers have strongly believed that *F. nucleatum* is prevalent and resident in high numbers during tooth brushing as well as a periodontal disease [99]. There are high chances that this transient oral fusobacteria may transmit through the circulatory system to spread CRA and CRC spots. Since *F. nucleatum* transition to tumor spots is a detrimental effect to the host [74], new avenues to block the enrichment of *F. nucleatum* in tumor sites or CRC would be therapeutically beneficial.

## 4. Bacteria-Mediated Cancer Treatment: Alternative to Surgery

Surgical treatment is the utmost general cancer therapy, and it has been practiced for several centuries [100]. Nevertheless, surgical procedure is not an actual treatment for metastatic conditions since they need radiation and chemotherapy [101]. The surgical procedure has lots of hitches that might offer incomplete elimination of tumor growth and possible reappearance [102]. The potential treatment of radiotherapy mostly affects tissue oxygen levels, however hypoxic environments occur in cancerous spots that result in failure of the treatment [101]. Moreover, the drug transition is therapeutically effective for chemotherapy; and deprived vasculature in cancer spots weakens drug delivery, which reduces the efficacy of the drugs, particularly in hypoxic and necrotic environments [102]. The usage of microbes in malignancy treatment has been practiced several times [102], and it is not well documented as an effective therapeutic tactic. Efficient knowledge and scientific progress have permitted generation of genetically programmed bacteria that drive harmless and effective application in cancer therapy. Bacteria-mediated cancer treatment facilitates facultative anaerobes that can able to live even in hypoxic and necrotic environments. It aids drug transition all over the tumor sites [103]. Nowadays, the sum of available bacterial therapy articles has been swiftly improved, in which the use of bacterial therapy of *Salmonella* has increased significantly [104].

## 5. Programmed Bacteria

Synthetic biology is dynamic to a new era of medicine through the genetic programming of living cells [105]. This transformative method allows for the creation of engineered systems and adding specificity and effectiveness that encompasses beyond the competences of molecular-based therapeutics [106,107]. One specific area of attention has been the programming of bacteria as therapeutic delivery systems to selectively discharge therapeutic cargos in vivo [105,106,108,109]. Chowdhury et al. [105] engineered a non-pathogenic *Escherichia coli* strain encoded with CD47nb, which elevates activation of tumor-infiltrating T cells, induces quick tumor regression, averts metastasis, and provides long-term survival in a syngeneic tumor mouse model. Harimoto et al. [110] screened *Salmonella typhimurium* strains expressing and carrying antitumor therapeutic molecules through various programmed gene circuits. In addition, these research groups have identified the candidates exhibiting noteworthy tumor reduction in a syngeneic mouse model [110]. This platform can be aided to identify the programmed diverse microbial species such as *Listeria monocytogenes*, *Proteus mirabilis*, and *Escherichia coli* in several host cell types [110].

Yoon et al. [111] also indicated that inherently adapted attenuated *Salmonella typhimurium*, that produces interferon-gamma (IFN-γ) as a tumoricidal agent, could elevate the therapeutic efficiency. In 1891, one of the greatest bone sarcoma surgeon named William B. Coley inoculated streptococcal organisms into a cancer patient, and those organisms successfully cured the patient of malignancy [104]. The anti-tumor activity of attenuated bacteria was achieved in various animal models. It has been achieved by antigen-specific tumor inhibition [112], reduced tumor mass [113,114], reduced proinflammatory stimulation [115,116], suppression of angiogenesis and pulmonary metastasized tumors [117], and eventually increases the survival time [118]. The list of programmed bacteria expressed as anti-tumor agents in various animal models are given in Table 2.

## 6. Mechanism of Tumoricidal Properties of Programmed Bacteria

Indeed, bacteria synthesize exotoxins through their type 1 secretion systems (T1SS) for their survival in the rigid environment [138]. T1SS is generally chaperone-dependent machinery employing proteins expressed by *hly* and *tol* genes [113]. Clinically significant T1SS cargo comprises of proteins, polysaccharides, ions, and small molecules, which are termed as exotoxin. For instance, uropathogenic *E. coli* contains a virulence factor of exotoxin: a-hemolysin (HlyA). This set of exotoxin normally aids pores on the host cells and consequently has the capacity to break down blood cells as well as cancer cells [139]. The studies showed that T1SS machinery activates chimeric human prostate-specific antigen (PSA), which elevates CD8^+^ cell-mediated reactions against a mouse mastocytoma [135]. Employing T1SS machinery in recent investigations showed a reduction of cancerous proliferation when *hlyE* was secreted by recombinant *S. typhimurium* using arabinose-inducible [113] and hypoxia-inducible bacterial promoter [114] in syngeneic hosts, even though the nature of anti-tumor reaction performed mainly not reliant on CD8^+^ cells.

The promoter of recombinant *S. typhimurium* [131] established to synthesize Shiga toxin in the tumor microenvironment, which causes tumor necrosis. When *E. coli* expresses a chimeric heterologous bacterial toxin, Listeriolysin-O (LLO), it can be elevated CD8^+^ cell-mediated antitumor response [125]. LLO is an exotoxin secreted by the bacterium *Listeria monocytogenes*, similar to hemolysin of *E. coli*. Likewise, when *S. typhimurium* synthesized a chimeric heterologous antigen, CD8^+^ cell-mediated anti-tumor reactions can be aroused [112]. Based on this experimental methodology, anti-tumor responses were initiated and experiments were performed using nude mice to control experimental tumors [140,141]. This high-throughput framework may serve to accelerate synthetic biology for translational medicine and for understanding the host-microbe interactions in disease sites.

## 7. Targeting Programmed *F. nucleatum* Fap2 for Colorectal Cancer

According to current studies, the tumor microenvironment and feces samples of patients with CRC are enriched by *F. nucleatum* [98]. Therefore, *F. nucleatum* is projected as one of the risk factors in the commencement and progression of CRC. The most significant mechanisms of *F. nucleatum* participated in CRC carcinogenesis are virulence factors (FadA, Fap2, RadD), miR-21, immunomodulation (inhibitory receptors of NK cells and elevating myeloid-derived suppressor cells), and metabolism of bacteria [98,142]. Studies showed that a host polysaccharide and lectin of fusobacterium that explains CRC comprises the richness of fusobacterium [28]. The host epithelium sugars of Gal-GalNAc are generally expressed higher in CRC, which is recognized by binding with fusobacterial Fap2. Thus, targeting fusobacterial Fap2 or host epithelium polysaccharide of Gal-GalNAc may decrease fusobacteria count in the CRC. Enormous studies previously described that bacteria act as anti-tumorigenic or anti-cancer agents [27,107,142]. The entire bacterium or the immunogenic properties of peptides carried by the bacteria show an effective anti-tumorigenic potent in the animal models of various cancers [106,107]. In this context, the application of attenuated host strains resulting from mutagenesis, rDNA techniques could also be practiced in bacteria that aid to eliminate tumor colonization. Vector encoded siRNA and shRNAs that target oncogenic materials, induction of tumor suppressor genes, and immunogenic peptides could also be developed. These methodologies show that the genetically programmed *F. nucleatum* has promising anti-tumor activity against CRC.

## 8. Conclusions

*F. nucleatum* infects the oral cavity to the colon that interrelates with the host immune system, ultimately cause inflammation and tumor progression. *F. nucleatum* exhibited the ability to cause CRC by Fap2-Gal-GalNAc complex specifically. However, attenuated *F. nucleatum*-Fap2 prevent transmembrane cellular signals and avert tumorigenesis persuading mechanisms. The genetically programmed practice can be performed in fusobacterial Fap2 by mutagenesis, rDNA techniques, attenuation of oncogenic materials, stimulation of immunogenic peptides and tumor suppressor genes. Targeting programmed fusobacterial Fap2 may reduce fusobacterium count in the CRC and promotes a healthy colon. Thus, the development of genetically programmed *F. nucleatum* Fap2 is a promising anti-tumor activity that will provide more methods for bacteria-based cancer treatment.

## Figures and Tables

**Table 1:** Detection of *F. nucleatum* in colorectal cancer (CRC) patients.

Sample Tested	Total Number of Clinical Samples	Detection Method	Positive Percentage	References
FFPE tissue	6	16S rRNA	32	[32]
FFPE tissue	6	16S rRNA	100	[33]
FFPE tissue	8	16S rRNA	100	[34]
Feces	14	qPCR	57	[35]
FFPE tissue	31	16S rRNA	10	[36]
FFPE tissue	37	16S rRNA	9	[37]
FFPE tissue	44	16S rRNA	100	[38]
FFPE tissue	46	16S rRNA	100	[39]
FFPE tissue	46	16S rRNA	54	[40]
FFPE tissue	47	16S rRNA	32	[41]
FFPE tissue	52	16S rRNA	77	[42]
Feces	72	qPCR	64	[43]
FFPE tissue	97	16S rRNA	72	[14]
Frozen tissue and FFPE tissue	101	FISH and FQ-PCR	87	[44]
Genomic DNA	149	qPCR	74	[45]
Feces	158	ddPCR	54	[46]
FFPE tissue	309	qPCR	34	[47]
FFPE tissue	511	qPCR	9	[48]
FFPE tissue	504	qPCR	56	[49]
FFPE tissue	598	qPCR	13	[26]

Abbreviation: ddPCR: droplet digital polymerase chain reaction; FFPE: formalin-fixed paraffin-embedded; FISH: fluorescence in situ hybridization; rRNA: ribosomal ribonucleic acid; FQ-PCR: fluorescent quantitative polymerase chain reaction; qPCR: quantitative real-time polymerase chain reaction.

**Table 2:** Programmed bacteria expressed as anti-tumor agents in various animal models.

Bacterial Species	Agent(s)	Host	Origin of the Tumor	Tumor(s)	Effector(s)	Results	References
*S. typhimurium*	*Bacterial antigen* *S. typhimurium* secreting *L. monocytogenes* Iap_217–225_ (Lm-p60)	BALB/c	Bones	WEHI-164 (Fibrosarcoma) cells expressing Lm-p60	CD8^+^ cell-mediated	Antigen-specific tumor inhibition	[112]
*S. typhimurium*	*Bacterial toxin* *S. typhimurium* secreting HlyE	BALB/c	Breast	CT-26, 4T1	Not reported	Reduction in tumor mass	[113,114]
*S. typhimurium*	Birc5 (Survivin)	C57BL/6	Lungs	D121	CD8^+^ cell-mediated	Suppression of angiogenesis and pulmonary metastasized tumors	[117]
*S. typhimurium*	BIRC5 shRNA NDUFA13 (GRIM-19)	Nude Mice	Larynx, prostate	Hep-2 (Laryngeal cancer) DU145 (PC-Xenograft)	Apoptosis	Tumor growth reduced	[119,120]
*S. typhimurium*	BIRC5 shRNA TNFSF15 (VEGI)	Nude mice	Breast	MDA-MB-231 (BC-Xenograft)	Apoptosis	Tumor growth reduced	[121]
*S. typhimurium*	ccl21	BALB/C	Breast	D2F2, CT-26	CD4^+^ and CD8^+^ cell mediated	Tumor-limited inflammatory reaction with a substantial reduction in tumor burden	[115]
*S. typhimurium*	CTAG1B (NY-ESO-1)	BALB/c	Skin	CMS5 cells expressing human NY-ESO-1	CD8^+^ cell-mediated	NY-ESO-1-positive tumors are eliminated	[122]
*S. typhimurium*	*Cytokine* ccl21	C57BL/6	Lungs	D121 (LC-Syngeneic)	CD8^+^ cell mediated	Suppression of angiogenesis and growth of pulmonary metastasized tumors	[117]
*S. typhimurium*	*Death inducer S. typhimurium* secreting murine Fasl	BALB/c	Breast, colon	CT-26 D2F2 (BC-Syngeneic)	Neutrophils	Reduction in tumor mass	[123]
*S. typhimurium*	Diablo/Trail	BALB/c C57BL/6	Liver, spleen, kidney	4T1 LL/2 (LC-Syngeneic) B16F10 (Melanoma)	Apoptosis	Tumor growth inhibition with prolonged survival	[124]
*E. coli*	*E. coli* expressing LLO	C57BL/6	Blood	MBL2 (Leukemia-Syngeneic) TRAMP-C (PC-Syngeneic)	CD8^+^ cell-mediated	Reduction in tumor mass	[125]
*S. typhimurium*	Growth inhibitor(s) Bcl2 shRNA	C57BL/6	Liver, spleen, skin	B16F10	Apoptosis	Survival time of tumor-bearing mice Prolonged Complete tumor regression not observed	[126]
*S. typhimurium*	HPV E6 shRNA TP53	Nude mice	Cervix	SiHa	Apoptosis	Tumor growth reduced	[127]
*S. typhimurium*	IL 18	C57BL/6	Skin	B16F1A (Melanoma)	Not reported	Increased survival time	[118]
		BALB/C	Skin, colon	D2F2, CT-26	Granulocyte, NK, CD4^+^, CD8^+^ cell mediated	Reduced tumor growth and pulmonary metastases	[111]
*S. typhimurium*	IL2	C57BL/6	Liver	MCA-38 (Adenocarcinoma Syngeneic)	NK cells	Hepatic metastases reduced	[116]
		BALB/C	Bone, lungs	K7M2 (Osteosarcoma –Syngeneic)	NK cells	Pulmonary metastases reduced compared to saline control	[128]
*S. typhimurium*	IL4	C57BL/6	Skin	B16F1A (Melanoma)	Not reported	Increased survival time	[118]
	MDM2 shRNA TP53-Cisplatin	Nude Mice	Prostate	PC3	Apoptosis	Tumor growth reduced	[129]
*S. typhimurium*	*S. typhimurium* secreting murine Trail	BALB/c	Breast	4T1	Apoptosis	Tumor growth reduced	[130]
*S. typhimurium*	*S. typhimurium* secreting Stx2	Nude Mice	Skin, colon	B16, HCT116, HeLa	Necrosis	Reduction in tumor mass	[131]
*S. typhimurium*	Stat3 shRNA	C57BL/6	Bone	H22	Apoptosis and CD8^+^ cell mediated	Tumor growth reduced	[132]
*S. typhimurium*	Stat3 shRNA Col18A1Endo	C57BL/6	Prostate	RM1	Apoptosis	Tumor growth reduced	[133,134]
*S. typhimurium*	Target antigen KLK3 (PSA)	DBA/2	Prostate	P815 cells expressing human PSA	CD8+ cell-mediated	Direct i.m. DNA vaccination was better than *Serovar typhimurium*-delivered immunogen	[135]
*S. typhimurium*	Tnfsf14 (LIGHT)	BALB/C	Breast	D2F2, CT-26	NK, CD4^+^, CD8^+^ cell- mediated	Primary and metastatic tumor growth inhibited	[136]
*S. typhimurium*	Vegfr2 (Kdr or Flk1) full-length Protein	BALB/c C57BL/6	Skin	CT-26 B16G3.26 (Melanoma) D121 MC-38 (CRC-Syngeneic)	CD8^+^ cell-mediated	Microvessel destruction retarded tumor growth and metastases. Healing of skin wounds slightly delayed. Immunological memory persisted at 120 days post-immunization	[137]
